# An interesting journey of an ingested needle: a case report and review of the literature on extra-abdominal migration of ingested Foreign bodies

**DOI:** 10.1186/1749-8090-6-77

**Published:** 2011-05-26

**Authors:** Zeynep Ozkan, Metin Kement, Ahmet B Kargı, Zafer Censur, Fazli C Gezen, Selahattin Vural, Mustafa Oncel

**Affiliations:** 1 General Surgery Department, Kartal Education and Research Hospital, Istanbul, Turkey; 2 Thoracic Surgery Department, Kartal Education and Research Hospital, Istanbul, Turkey

**Keywords:** Foreign body, migration, pneumotomy

## Abstract

Swallowed foreign bodies encounter a major problem especially in children, but fortunately they mostly do not cause any related complication and are easily passed with the stool. In this paper, an interesting journey of a needle is presented. A 20-year old female admitted to our emergency service after she had swallowed a sewing machine needle, which is initially observed in the stomach in the plain abdominal radiography. During the follow-up period, the needle traveled through bowels, and surprisingly was observed in the left lung on 10^th ^day of the follow-up. It was removed with a thoracotomy and pneumotomy under the fluoroscopic guidance. The postoperative period was uneventful and the patient was discharged from the hospital on the day 5. We also review the literature on interesting extra-abdominal migrations of swallowing foreign bodies.

## Background

The foreign body ingestion occurs usually in children. Although it is detected rarely in adults, prisoners, mentally retarded people and young girls with turban in Islamic countries are commonly affected [1-3]. Foreign bodies generally pass spontaneously through the gastrointestinal tract (GI tract) and do not result in any complications, but very sharp or pointed objects may cause perforations along the gastrointestinal tract. In addition, retained foreign bodies may cause gastrointestinal erosions and abrasions, which result in bleeding. The rate of complication from foreign body ingestion is estimated less than 1%. Complications due to foreign bodies in the stomach and small intestine typically involve perforation associated with peritonitis. Foreign bodies account for 15% to 35% of all bowel perforations. These cases require surgical intervention. Although migration of foreign bodies from esophagus to mediastinum and thorax may lead to very serious complications including pneumomediastinum, mediastinitis, pneumothorax, pericarditis, cardiac tamponade, or even horrific lethal vascular injuries to the aorta or pulmonary vasculature, migration of foreign bodies from the colon to the lung is not reported before [4-8].

In this report, we present an interesting journal of an ingested sewing machine needle which migrated from the transverse colon to the lung in a young woman. We also review the literature on interesting extra-abdominal migrations of swallowing foreign bodies.

### Case

**Figure 1:**
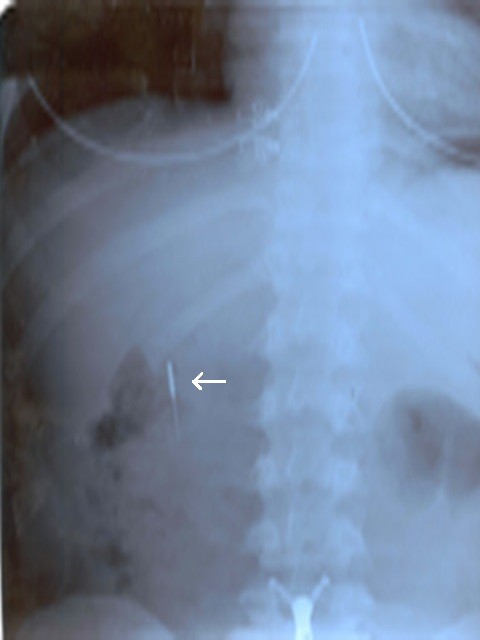
**A plain abdominal radiography (PAR) revealed a needle located in the upper abdomen**.

**Figure 2:**
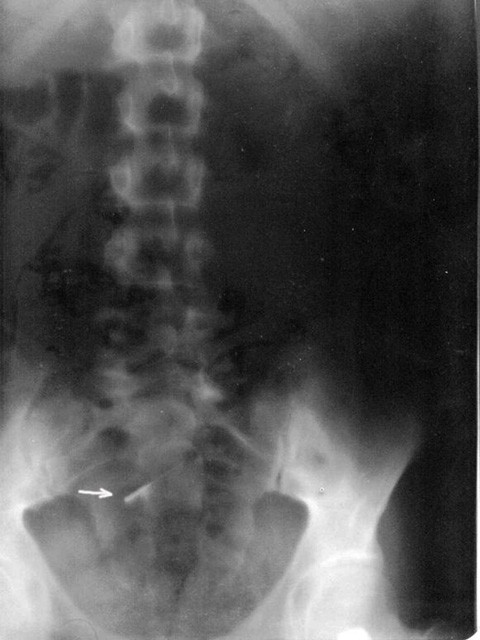
**The needle had passed to the terminal ileum and transverse colon**.

**Figure 3:**
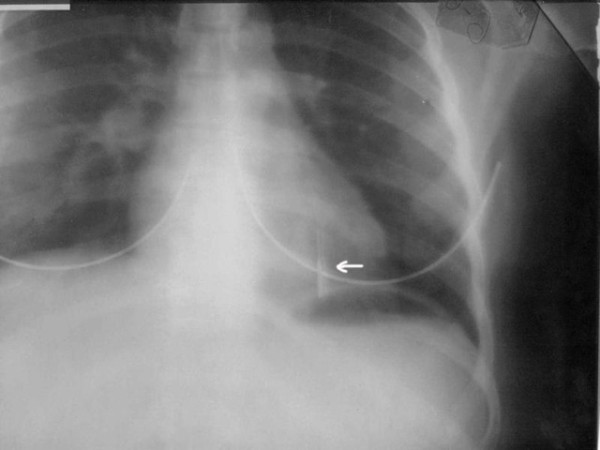
**PAR showed that needle migrated into the thorax**.

**Figure 4:**
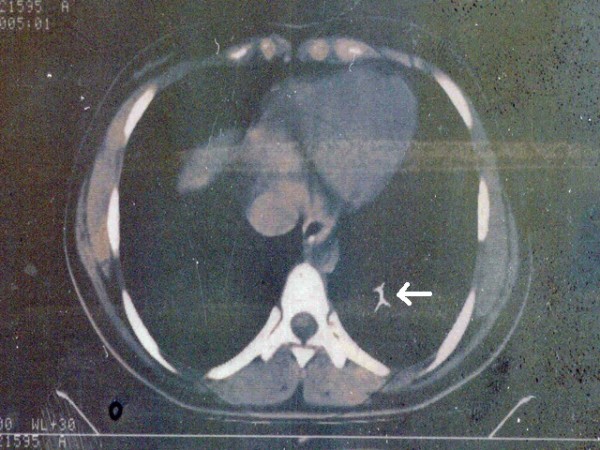
**An emergent computarized tomography confirmed that the needle was located in the lower lobe of the left lung**.

A 20-year old female was admitted to our emergency service immediately after accidental swallowing of a sewing machine needle. On admission, she had no symptoms such as abdominal pain, vomiting or dysphagia. A plain abdominal radiography (PAR) revealed a needle located in the upper abdomen (Figure 1). A fiber diet was prescribed and a daily routine out-patient follow-up with PAR's was planned. The two PAR's taken on the days 3 and 7 showed that the needle had passed to the terminal ileum and transverse colon (Figure 2). However, on the day 10, PAR showed that needle migrated into the thorax (Figure 3). She did not have any symptoms or signs of peritonitis. An emergent computerized tomography (CT) confirmed that the needle was located in the lower lobe of the left lung (Figure 4). Also, there was no intestinal contrast leakage in CT. Therefore, it was decided to perform an emergent thoracotomy. Fluoroscopy and finger palpation were used to verify the exact location of the needle during the operation and the needle was removed after pneumotomy. The postoperative period was uneventful and the patient discharged from the hospital on the day 5.

## Discussion

The incidence of foreign body ingestions is unknown. The most common causes of foreign body ingestion are accidental swallowing of objects. Children usually put any object they find into their mouths and may accidentally swallow them. In healthy adults, accidental swallowing often involves toothpicks, dentures and turban pins. Psychiatric patients may swallow a wide variety of objects, including large and bizarre items. Although the majority of foreign bodies pass harmlessly through the GI tract and conservative management is generally recommended, 10% to 20% of them will require non-operative intervention such as endoscopy, and approximately 1% of them will require surgery [9-11]. An estimated 1500 deaths occur annually from foreign body ingestion in USA [12].

A foreign body lodged in the gastrointestinal tract may cause local inflammation leading to pain, bleeding, scarring and obstruction, or it may erode through the GI tract. The site of perforation due to foreign bodies appears to be variable in the GI tract. Although McManus et al. identified ileocecal region as the most common site of perforation secondary to foreign body ingestion [13], duodenum has been reported as the most common site by Spitz et al. and Gracia et al. [14,15]. Foreign body ingestions necessitate careful and continued observation due to the possibility of serious complications.

Children and the mentally impaired, or the psychiatric patients may present with refusal to eat, vomiting, choking, drooling, wheezing, blood-stained saliva, or respiratory distress. Erythema, tenderness, or crepitus in the neck may be present with oropharyngeal or esophageal perforation. The abdomen should be examined for clinical evidences of peritonitis. These conditions will require emergent surgical intervention. Ventilation, airway compromise and the risk of aspiration should also be assessed. If the swallowed object is radio-opaque, a single frontal radiograph that includes the neck, chest, and entire abdomen is usually sufficient to locate the object. The plain radiography is effective in localizing most of radio-opaque objects [16]. CT scan or MRI is rarely indicated but may enhance the detection of foreign bodies or complications (e.g., perforations, migrations) in special cases.

Migration of foreign bodies from the abdomen or pelvis to the lung is very rare but well-defined entity. Diagnostic catheters, venous shunts and bullets have been reported in this context, but differently from our case, most of them have migrated hematogenously [17-19]. To our knowledge, our case is unique in that we have described a foreign body migration from the transverse colon to the lung parenchyma. Interestingly, that migration did not have lead peritonitis and the patient was able to manage without the need for a laparotomy. We think, the perforation in the transverse colon wall was too small to cause a significant bowel leakage. Also, the route of the journey after transverse colon is not certain. In our opinion, there are two possibilities for the needle to pass thorax: either, it penetrated the diaphragm or passed through esophageal hiatus.

In reviewing the literature on extra-abdominal migration of swallowing foreign bodies, Macchi at al. reported a case of a 48-year-old man with esophageal perforation, mediastinitis, and evidence of perforation of the ascending aorta during surgical drainage of the mediastinum. They reported finding a fish bone under the aortic arch at autopsy [5]. Kunishige et al. presented a 79-year-old woman who had referred to hospital with chest pain after swallowing a fish bone. The bone had been removed by esophagoscopy. Eleven days later she had presented because of hematemesis. Computed tomography and angiography had confirmed a diagnosis of esophageal perforation leading to mediastinitis and the presence of an infected pseudoaneurysm. The infected pseudoaneurysm had been completely resected [6]. Cekirdekci et al. and Vesna et al. reported two different cases with cardiac tamponade due to migration of sewing needle from the esophagus [7]. On the contrary, Graffstädt et al. presented a journey of a wandering needle from bronchus to intestine in a 14-year-old girl. The needle had been excreted naturally on the third day [20]. Ozsunar et al. presented an interesting unique case in which a needle had been accidentally swallowed and then migrated into the vertebral body [21]. Chen et al. reported a 50-year-old woman who had been diagnosed with thyroid abscess secondary to a swallowing fish bone [22].

As a conclusion, an ingested foreign body infrequently causes severe problems, however complications such as perforation and migration should be always keep in mind and close follow up should be done. In addition, we have to be certain to detect that foreign bodies have left the body.

## Consent

Written informed consent was obtained from the patient for publication of this case report. A copy of the written consent is available for review by the Editor-in-Chief of this journal.

## Competing interests

The authors declare that they have no competing interests.

## Authors' contributions

ZO, MK and ZC were involved in patient care. ZO and MK reviewed the literature and wrote the manuscript. ABK performed operation. CG and MO supervised the manuscript. All authors read and approved the final manuscript.

## References

[B1] Stack LB Munter DW Foreign bodies in the gastrointestinal tract Emerg Med Clin North Am 1996 14 493 521 10.1016/S0733-8627(05)70264-9 8681881

[B2] Cheng W Tam PK Foreign-body ingestion in children: experience with 1,265 cases J Pediatr Surg 1999 34 1472 1476 10.1016/S0022-3468(99)90106-9 10549750

[B3] Conway WC Sugawa C Ono H Lucas CE Upper GI foreign body: an adult urban emergency hospital experience Surg Endosc 2007 21 455 460 10.1007/s00464-006-9004-z 17131048

[B4] Ghimire A Bhattarai M Kumar M Wakode PT Descending necrotizing mediastinitis: a fatal complication of neglected esophageal foreign body Kathmandu Univ Med J (KUMJ) 2007 5 98 101 18603994

[B5] Macchi V Porzionato A Bardini R Parenti A De Caro R Rupture of ascending aorta secondary to esophageal perforation by fish bone J Forensic Sci 2008 53 1181 1184 10.1111/j.1556-4029.2008.00815.x 18643867

[B6] Kunishige H Myojin K Ishibashi Y Ishii K Kawasaki M Oka J Perforation of the esophagus by a fish bone leading to an infected pseudoaneurysm of the thoracic aorta Gen Thorac Cardiovasc Surg 2008 56 427 429 10.1007/s11748-008-0266-3 18696212

[B7] Cekirdekci A Ayan E Ilkay E Yildirim H Cardiac tamponade caused by an ingested sewing needle. A case report J Cardiovasc Surg (Torino) 2003 44 745 746 14735038

[B8] Vesna D Tatjana A Slobodan S Slobodan N Cardiac tamponade caused by migration of a swallowed sewing needle Forensic Sci Int 2004 139 237 239 10.1016/j.forsciint.2003.10.013 15040923

[B9] Webb WA Management of foreign bodies of the upper gastrointestinal tract: Update Gastrointest Endosc 1995 41 39 51 10.1016/S0016-5107(95)70274-1 7698623

[B10] Nandi P Ong GB Foreign body in the esophagus: Review of 2394 cases British Journal of Surgery 1978 65 5 9 10.1002/bjs.1800650103 623968

[B11] Vizcarrondo FJ Brady PG Nord HJ Foreign bodies of the upper gastrointestinal tract Gastro-intest Endosc 1983 29 208 210 10.1016/s0016-5107(83)72586-1 6618118

[B12] Stack LB Munter DW Foreign bodies in the gastrointestinal tract Emerg Med Clin North Am 1996 14 493 521 10.1016/S0733-8627(05)70264-9 8681881

[B13] MacManus JE Perforations of the intestine by ingested foreign bodies Am J Surg 1941 53 393 10.1016/S0002-9610(41)90652-9

[B14] Spitz L Management of ingested foreign bodies in childhood Br Med J 1971 4 469 472 10.1136/bmj.4.5785.469 5125285 PMC1799648

[B15] Gracia C Frey CF Bodai BI Diagnosis and management of ingested foreign bodies: A Ten Years Experience Annals of emergency medicine 1984 13 159 10.1016/s0196-0644(84)80380-7 6689853

[B16] Suita S Ohgami H Nagasaki A Yakabe S Management of pediatric patients who have swallowed foreign objects Am J Surg 1989 55 585 2774368

[B17] Gupta AK Dogra VS Ahmad I DelBalso AM Missile emboli to the pulmonary artery [letter] Am J Emerg Med 1997 15 213 214 10.1016/S0735-6757(97)90112-1 9115534

[B18] Doughty IM David TJ Migration of fine bore Silastic catheter to pulmonary artery [letter] Arch Dis Child 1994 70 451 8017976 10.1136/adc.70.5.451 PMC1029844

[B19] Planas R Domenech E Montana X Pilar Rodriguez M Rodriguez N Cabré E Gassull MA Rupture and migration of the venous segment of LeVeen shunt: an unreported complication Am J Gastroenterol 1993 88 1101 1103 8317413

[B20] Graffstädt H Dieckow B Grüber C Stöver B Niggemann B Christmas surprise: the unnoticed journey of a needle-from bronchus to intestine Respir Med 2005 99 1600 1602 10.1016/j.rmed.2005.03.022 16291081

[B21] Ozsunar Y Tali ET Kilic K Unusual migration of a foreign body from the gut to a vertebral body Neuroradiology 1997 40 673 674 10.1007/s002340050663 9833900

[B22] Chen CY Peng JP Esophageal fish bone migration induced thyroid abscess: case report and review of the literature Am J Otolaryngol 2010 4 29 10.1016/j.amjoto.2010.02.006 20434801

